# Reconciling Safety and Safeguarding in Health and Social Care: Implications for Just Culture

**DOI:** 10.3390/healthcare13070690

**Published:** 2025-03-21

**Authors:** Siobhán E. McCarthy

**Affiliations:** Graduate School of Healthcare Management, RCSI University of Medicine and Health Sciences, D02 YN77 Dublin, Ireland; smccarthy@rcsi.ie

**Keywords:** just culture, safety, safeguarding

## Abstract

Facilitating a just response to staff involved in patient safety events is complex, with varying perceptions of safe behaviour and practice across settings. This viewpoint paper explores the challenges of developing a just culture, particularly in safeguarding situations involving peer-to-peer harm. It argues that established just culture principles, such as balancing staff and organisational accountability and using After Action Review (AAR) debriefs, need to be tailored to these contexts. In particular, organisational accountability is paramount in safeguarding situations, especially where individuals do not have the capacity to understand or intend their behaviours. Furthermore, AARs are inappropriate incident responses for serious aggression, violence, and abuse cases. To counter this, a consistent AAR practice can be valuable for preventative learning when applied to the service user care journey and comprehensive incident learning responses. The incorporation of social workers, service users, and families can help promote learning and the prevention of events. Finally, this paper emphasises the need for consistency in core safety principles across settings and the need to tailor just cultural principles to particular contexts. Future research on the role of AAR in diverse settings is recommended.

## 1. Introduction

Just culture, discerning what is acceptable behaviour and an appropriate response, is a challenging goal for health and social care systems [1]. In theory, when an unintended patient safety event occurs, a just culture ensures that staff are not punished for actions commensurate with their experience and training but also ensures gross negligence and destructive acts are not tolerated [2]. Requirements for a just culture include speaking up, support for staff involved in events, an intolerance of unacceptable behaviour, a systems viewpoint on how errors happen, and human-centred healthcare design [3]. The concept of just culture is attractive to healthcare professionals involved in patient safety events, as it shifts the focus away from individual blame for system errors towards learning and accountability. In theory, a just culture provides a balance between individual and organisational accountability and provides opportunities for open and inclusive conversations when incidents occur [4]. Implementation requires just culture education, as well as leadership support and a plan for sustainability [5]. However, barriers to just culture are numerous [6]. Different professional groups and organisational contexts may disagree on acceptable behaviour and appropriate responses [6,7,8]. Additionally, hindsight bias skews judgment depending on the severity of the event’s outcome [7]. Across settings, the severity of the event outcome may become known across dramatically different time frames. For example, minutes in acute settings or years in residential or community care. Using the Irish health and social care system as an example, this paper discusses how safety and safeguarding are defined across the acute, residential, and community sectors in Ireland, as well as the feasibility of realising a just culture within these settings. Finally, the potential of After-Action Review (AAR), a debriefing tool, to help healthcare teams develop a just culture and come to a shared understanding of acceptable behaviour and appropriate response is discussed.

## 2. Safety and Safeguarding in the Irish Health and Social Care System

In healthcare, safety is often defined as freedom from accidental injury or harm associated with healthcare [9]. Common adverse events, such as healthcare-associated infections, falls, pressure ulcers, and surgical site infections, are perceived to arise not from a patient’s medical condition but from their healthcare management [10]. Common preventable adverse outcomes include increased hospital stay, disability and death, as well as psychological harm to families and caregivers and second-victim impact on staff [9]. Second-victim impact comprises the psychological, physical and professional trauma experienced by staff who are unintentionally involved in a patient safety event [11].

Despite efforts to the contrary, adverse patient safety events are increasing worldwide [9]. In Ireland, two national patient chart reviews indicated that approximately one in eight admissions to acute hospitals were associated with an adverse event with little improvement between 2009 and 2015 [12,13]. Factors affecting the growing incidence include the complexity of healthcare, ageing populations, and the continuing expansion of items considered a patient safety event [10]. In recent years, there has been a shift in focus from researching the prevalence of patient safety events to examining how well institutions and teams learn, engage in quality improvement, and manage their capacity [14,15,16]. For example, international patient safety research frameworks emphasise the need for real-time safety monitoring alongside the measurement of locally implementable and sustainable solutions [17,18,19,20]. This shift resonates with just cultural themes, i.e., with learning and improvement being a form of accountability for the majority of errors that arise from system factors [4]. Evidently, more disciplinary forms of response are required in the case of wilful violations.

More broadly, in health and social care in Ireland, there is a parallel shift towards designing adult safeguarding strategies that integrate with overall service user safety policies [21,22]. How these shifts resonate with realising a just culture is, as yet, unclear, particularly in situations of peer-to-peer abuse [23]. First, safeguarding is the term used to ensure that individuals in health and social care settings can live free from abuse [24]. Safeguarding is essential to high-quality health and social care [25]. It involves putting in place measures “to reduce the risk of harm, promote and protect people’s human rights and their health and wellbeing, and empower people to protect themselves” [25]. People with frailty due to age, a physical or intellectual disability, an acquired brain injury, a mental health condition or persons living under coercive control are considered most at risk of abuse [25]. Examples of common types of abuse include emotional, financial, physical, sexual, and organisational abuse (i.e., systematic poor care) as well as neglect [26]. In its 2023 annual report, the National Safeguarding Office (NSO) identified 22,082 alleged abuse types within 18,290 reported concerns [27]. Psychological and physical abuse were the most common types of abuse reported. For residents aged 65 years and over, 37% of concerns related to immediate family members, 33% to peers, and 20% to staff members [27]. Potentially, as an indication of the lack of assurance around the implementation of a just culture, the report recommended the need to further explore “resident-to-resident aggression” to better understand the contributory factors and protective responses required [27]. The report stated that peer-to-peer abuse may not always be perceived as a safeguarding concern [27]. A subsequent 2024 research report conducted by the Health Information & Quality Authority (HIQA) on behalf of Safeguarding Ireland identified the need to differentiate between peer-to-peer aggression and peer-to-peer abuse. Peer-to-peer abuse was then defined as “offensive, aggressive and intrusive verbal, physical, sexual, and material interactions between service users that in a community setting would likely be unwelcome and potentially cause physical or psychological distress or harm to the recipient/victim” but experts could not agree on when aggression progresses to abuse [23]. Criteria for conducting this assessment included whether the person acted on purpose and was able to understand their actions, the feelings of the victim, the behavioural needs of the people involved, and the settings in which the incident took place [23]. Due to this emerging differentiation, guidance is required to assess the organisational antecedents of each of these behaviours, especially in contexts where people lack the capacity to understand or intend their behaviours. Future awaited policy aligned with these terms will better assist the safeguarding of residents and provide the opportunity to apply a just response. The current ambiguity risks the safety of those who do not have the capacity to choose where they live despite exposure to repeated peer aggression or abuse, or lack of behavioural support.

Clearly, challenging or abusive behaviour in health and social care may not always be perceived in a vigilant manner. There may be tendencies to medicalise, accept, or ignore these forms of behaviour and avoid organisational responsibility. For example, a recent 2025 HIQA review found that an intellectual disability service with centres across Ireland had restrictive practices, poor management of safeguarding incidents, inadequate staffing arrangements, and poor behavioural support for residents [28]. Some of the residents reported feeling unsafe in their homes due to the ongoing aggressive behaviour of other residents. Furthermore, while complaints were made, concerns remained unresolved [28]. Evidently, not knowing or initiating the appropriate response to behaviours, which in other settings may be perceived as criminal behaviour, is a challenge to just culture and, ultimately, to the rights of all residents to live free from abuse. Implementing a just response based on prevention, accountability, and meeting peoples’ needs in real time should help ensure all are safeguarded. Variations in what is acceptable from setting to setting should not be permitted.

## 3. After Action Review as a Tool to Promote Just Culture

The Irish health and social care system has promoted AAR practice as a means to help teams come together to discuss patient safety and everyday events [15]. In AARs, discussion centres around four questions: what did we expect to happen, what actually happened, why was there a difference, and what have we learned [15]? Guidelines for facilitating AARs include respect, non-hierarchy, focus on learning and improvement, no blame, and representation of what was experienced [29]. These align with the characteristics of shared responsibility in a just culture, including withholding judgment, expressing diverse opinions, avoiding blame, team-reflective learning and identifying ways to improve [30]. AARs have formed part of incident management policy in the Irish healthcare system since 2018 and have been encouraged for use in acute and community settings [31]. AARs are recommended as a concise review option for minor or moderate incidents and may be used to debrief major incidents, complementary to other review approaches [32]. In AARs, there is a focus on equal and open participation and the entitlement of all to voice individual perspectives [15]. The establishment of AAR in the Irish healthcare system can be viewed as a challenge to the here-to-fore dominant approach to the treatment of safety issues outlined in Table 1. The discussion around mainstream patient safety has predominantly viewed it as a socio-technical issue, where individual behaviours, slips and lapses intersect with systems and infrastructure in a manner that can make patient safety events happen [33]. The challenge is that the socio-technical system has been dominated by a bio-medical model, often privileging medical and nursing perceptions of safety, whereas allied health and social work views have been less privileged [33]. Given the attempt to have non-hierarchy predominate the AAR conversation, AARs can be seen as an attempt to democratise discourse in patient safety, with all having an equal opportunity to influence the safety conversation, a shared mental model about safety, as well as follow-up actions.

It is noteworthy that when concerns are raised in residential settings, it is often social workers who are the first port of call to address issues. Yet, social workers operate under a high degree of uncertainty, requiring the need to balance safety with autonomy for human rights [34]. Social workers, like other healthcare professionals, will experience a second victim impact when care goes wrong, but how and when do errors become apparent? Are just responses simultaneously fair in the short and longer term, and what mechanisms can help social work teams uncover this? Thus, it is acknowledged that applying the principles of just culture is more straightforward in an acute medical setting than in a residential setting. This is due to the complexity of relations among service users, their range of care providers, and the large amount of time spent living in care settings. However, creating a culture of AAR practice, including reviewing the chosen responses to these issues (i.e., undertaking an AAR of the incident response), can promote a shared understanding of the fairness and effectiveness of responses over time.

Furthermore, it is clear the just culture principle of balancing individual and organisational accountability is difficult to apply to situations of peer-to-peer aggression and abuse, where capacity issues are involved. This principle needs to be tailored to fundamentally safeguard service users affected by these behaviours while also balancing the provision of behavioural support to those exhibiting these behaviours; organisational responsibility is important to manage capacity and resources to ensure all are safeguarded.

It is also clear that an AAR is not an appropriate incident response in situations of peer-to-peer abuse or violence, and comprehensive risk assessments, investigations, and interventions are needed [31]. However, proactively applying AARs to components of care journeys and pathways, rather than isolated events [35], may help foster a culture of ongoing team reflection in residential and home care settings. Real-time safety analysis using AARs can allow for a restorative approach by focusing on both the positive and challenging aspects of care [29]. The benefit of consistent AAR practice may be preventative, particularly regarding peer-to-peer aggression escalating towards abuse. For minor events, informal AARs (i.e., brief, facilitator-free reviews without documentation) have been found to be more readily implementable and accepted by staff [29].

AARs are primarily a mechanism for staff debriefing, and service user concerns are typically met through a separate process called open disclosure [32]. However, consistent AAR practice in settings could enable the potential for service users and their families to become involved in AAR conversations, where appropriate. Informally using AAR questions with service users and families can initiate their involvement and promote a restorative focus by understanding their perspectives and utilising these to co-design improvements. The inclusion of a range of persons affected, without compromising staff willingness to engage in AARs or overwhelming service users, will enhance learning and lead to more tailored improvements [36].

## 4. Conclusions

A just culture may be more difficult to operationalise in residential settings, particularly where safeguarding concerns are apparent from peer-to-peer harm. Implementing a just culture remains a challenge universally, and tools like AARs are needed to help democratise the conversation about what is safe and what is to be learned. Where behaviours are found to be destructive and wilfully violate safe practices, AARs are not the appropriate response mechanism, and a comprehensive review is required. Nevertheless, should these be discovered in an AAR, AAR facilitators and organisers must be able to transfer these issues to an appropriate authority. Promoting just culture is as much about preventing adverse events and safeguarding concerns as it is about responding to issues when they arise. Consistent AAR practice, particularly during the patient’s journey, may help prevent and facilitate responses to safeguarding concerns, thereby promoting a just culture. Research is needed to compare and contrast the role of AAR in safety management and in the promotion of a just culture across various settings.

## Figures and Tables

**Table 1:** Comparison of the traditional and emerging focus of safety learning in health and social care.

	Traditional Focus of Safety Learning	Emerging Focus of Safety Learning
Areas of health and social care	Acute Care	Acute, primary and social care Integrated care
Safety concerns	Patient safety	Patient and staff safety Safeguarding Integration of safety and safeguarding
	Individual events	Care journeys and pathways over time and across settings
Learning approach	Investigative and hierarchical focus	Graded learning response options from investigative to facilitative non-hierarchical approaches
	Lengthy time frame	Immediate facilitated learning in parallel to lengthy reviews
	Individual learning	Team learning in real time after the occurrence of events
	Comprehensive learning reports	Learning linked to formal quality improvement processes in addition to comprehensive learning reports
Professional perspectives	Socio-technical focus with primacy of medical and nursing views	Socio-technical focus with equality of multi-disciplinary views
Patient and family perspectives	Closed to patients and families until end of process	Inclusive of patients and families along all elements of the learning pathway
Accountability style	Individual blame	Balance of individual and organisational accountability
	Little systemic change	Quality improvement culture
